# Aloe-derived vesicles enable macrophage reprogramming to regulate the inflammatory immune environment

**DOI:** 10.3389/fbioe.2023.1339941

**Published:** 2023-12-21

**Authors:** Hao Zhou, Ke Peng, Jun Wang, Yang Wang, Jia-Jia Wang, Shi-Kun Sun, Mai-Qing Shi, Jun Chen, Fu-Hai Ji, Xu Wang

**Affiliations:** ^1^ Department of General Surgery, The First Affiliated Hospital of Soochow University, Suzhou, Jiangsu, China; ^2^ Department of Anesthesiology and Institute of Anesthesiology, The First Affiliated Hospital of Soochow University, Suzhou, Jiangsu, China; ^3^ Department of Anesthesiology, The First Affiliated Hospital of Soochow University, Suzhou, Jiangsu, China; ^4^ Department of Intensive Care Medicine, The First Affiliated Hospital of Soochow University, Suzhou, Jiangsu, China; ^5^ Department of Pulmonary and Critical Care Medicine, The First Affiliated Hospital of Soochow University, Suzhou, Jiangsu, China; ^6^ Department of Cardiology, The First Affiliated Hospital of Soochow University, Suzhou, Jiangsu, China

**Keywords:** pneumonia, extracellular vesicle nanovesicles, aloe, macrophages reprogramming, immunoregulation

## Abstract

**Introduction:** Bacterial pneumonia poses a significant global public health challenge, where unaddressed pathogens and inflammation can exacerbate acute lung injury and prompt cytokine storms, increasing mortality rates. Alveolar macrophages are pivotal in preserving lung equilibrium. Excessive inflammation can trigger necrosis in these cells, disrupting the delicate interplay between inflammation and tissue repair.

**Methods:** We obtained extracellular vesicle from aloe and tested the biosafety by cell viability and hemolysis assays. Confocal microscopy and flow cytometry were used to detect the uptake and internalization of extracellular vesicle by macrophages and the ability of extracellular vesicle to affect the phenotypic reprogramming of macrophages *in vitro*. Finally, we conducted a clinical feasibility study employing clinical bronchoalveolar lavage fluid as a representative model to assess the effective repolarization of macrophages influenced by extracellular vesicle.

**Results:** In our study, we discovered the potential of extracellular vesicle nanovesicles derived from aloe in reprograming macrophage phenotypes. Pro-inflammatory macrophages undergo a transition toward an anti-inflammatory immune phenotype through phagocytosing and internalizing these aloe vera-derived extracellular vesicle nanovesicles. This transition results in the release of anti-inflammatory IL-10, effectively curbing inflammation and fostering lung tissue repair.

**Discussion:** These findings firmly establish the immunomodulatory impact of aloe-derived extracellular vesicle nanovesicles on macrophages, proposing their potential as a therapeutic strategy to modulate macrophage immunity in bacterial pneumonia.

## Introduction

Bacterial pneumonia caused by *Streptococcus pneumoniae*, *Staphylococcus aureus*, Gram-negative rods, and *Acinetobacter* is a significant public health issue (Cilloniz et al., 2011; Torres et al., 2017; Braverman et al., 2022). This disease severely impacts the alveoli and distal bronchial tree in the lungs. Failure to eliminate the pathogens and the associated inflammatory response can lead to acute lung injury, resulting in a high mortality rate, especially among children, the elderly, and individuals with compromised immune systems (Shi et al., 2020). Alveolar macrophages (AMs) play a vital role in lung immunity and tissue repair (Lambrecht, 2006; Hussell and Bell, 2014). Currently, the primary treatment for bacterial pneumonia involves antibiotics (Alvarez-Lerma, 1996; Ott et al., 2012). However, the widespread development of antibiotic resistance due to their extensive use in clinical treatment has led to treatment failures and exacerbated inflammation (Magiorakos et al., 2012). The increased inflammatory response causes non-apoptotic death of AMs, disrupting the homeostasis provided by these macrophages in terms of immunity and tissue repair (Gonzalez-Juarbe et al., 2015). The release of pro-inflammatory substances from necrotic cells triggers a more severe innate immune response, recruiting inflammatory monocytes–macrophages and neutrophils to the damaged sites, resulting in the secretion of a significant amount of pro-inflammatory cytokines like TNF-α, IFN-γ, IL-6, and IL-1β (Wen et al., 2020; Monteith et al., 2021; Zhang et al., 2021). This can lead to complications such as sepsis and cardiovascular disease (van der Poll et al., 2017). Therefore, controlling the inflammation levels in lung tissue becomes crucial.

Extracellular vesicles (EVs) are nanosized particles (ranging from 30 to 120 nm) that can be released from any cell, including both animal and plant cells. They carry a variety of substances such as DNA, RNA, proteins, and lipids, facilitating the exchange of important biomolecules and genetic information between different cells (Colombo et al., 2014; Peng et al., 2020; Dad et al., 2021; Xu et al., 2021; Boccia et al., 2022). This exchange can establish communication and influence cellular behavior between the same or different organisms (Dad et al., 2021). Due to the low immunogenicity and resistance to clearance by immune cells, EVs are efficient at delivering biomolecules and influencing cellular behaviors (Ju et al., 2013; Wang et al., 2014). Mammalian-derived EVs have been extensively studied and validated for intercellular communication, physical characteristics, and vesicle functions. In contrast, EVs from plant sources, although discovered earlier than their mammalian counterparts, have been relatively understudied in terms of their biological effects on the human body (Dad et al., 2021). Recent research has successfully demonstrated that EVs derived from grapes, grapefruits, ginger, and aloe contribute to tissue regeneration and inflammation relief (Ju et al., 2013; Wang et al., 2014; Zhang et al., 2016; Kim et al., 2021). Furthermore, plant-derived EVs have a lower immunological risk and fewer side effects than mammalian-derived vesicles, alleviating concerns related to potential animal or human pathogens (Dad et al., 2021). These characteristics suggest that plant-derived EVs hold significant potential for immune regulation.

In this research, we successfully isolated and purified EV nanoparticles from aloe. The analysis results indicate that aloe-derived extracellular vesicle nanoparticles exhibit typical extracellular vesicle morphology and size. They can polarize pro-inflammatory M1 macrophages into anti-inflammatory M2 macrophages, effectively mitigating the cytokine storm and lung alveolar tissue damage caused by the overactive immune response during pneumonia development. These results suggested that aloe-derived EV nanoparticles have significant potential for treating bacterial pneumonia.

## Results

### Preparation and characterization of EV_Aloe_

**FIGURE 1:**
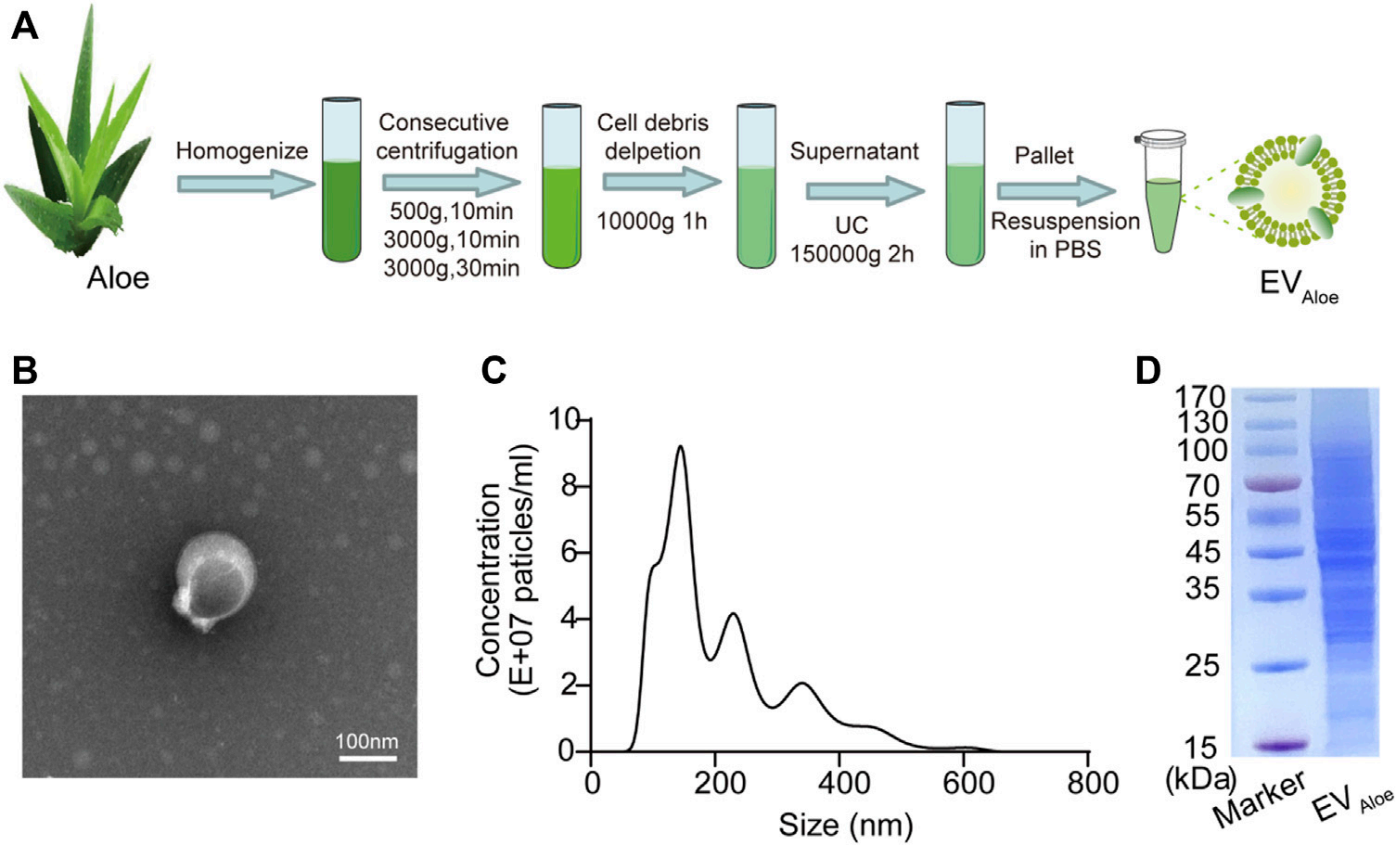
Fabrication and characterization of EV_Aloe._ **(A)** Isolation and preparation of EV_Aloe._ EV_Aloe_ could be isolated and prepared by a series of centrifugations, including ultracentrifugation and sucrose gradient ultracentrifugation. **(B)** TEM image of EV_Aloe_. EV_Aloe_ harvested from the sucrose density gradient (45%) was characterized by TEM. Scale bar: 100 nm. **(C)** Size distribution of EV_Aloe_ was measured by NTA. **(D)** SDS-PAGE analysis of the protein components of EV_Aloe_. The proteins in EV_Aloe_ were analyzed via 10% SDS-PAGE.

To investigate the properties of aloe-derived EV nanoparticles, we isolated and purified EV_Aloe_ from the aloe-homogenized juice (defined as EV_Aloe_) by consecutive centrifugation and ultracentrifugation (Figure 1A), and subsequent transmission electron microscopy (TEM) examination (Figure 1B) and nanoparticle tracking analysis (NTA) (Figure 1C) revealed that the EV_Aloe_ exhibited a classic cup-shaped spherical structure, with an average diameter of 144.5 ± 2.8 nm. The purified EV_Aloe_ was quantified using a micro-bicinchoninic acid (BCA) protein analysis kit. The results indicated a high abundance of EV nanoparticles in aloe (approximately 500 mg/kg), suggesting that aloe can generate a significant amount of EV nanoparticles. Furthermore, we conducted a duplicate analysis of the protein composition of the purified EV_Aloe_ using sodium dodecyl sulfate–polyacrylamide gel electrophoresis (SDS-PAGE) (Figure 1D). The findings revealed a plethora of proteins within the EV_Aloe_ that potentially possess immune-modulating capabilities.

### EV_Aloe_ shows a favorable safety test

**FIGURE 2:**
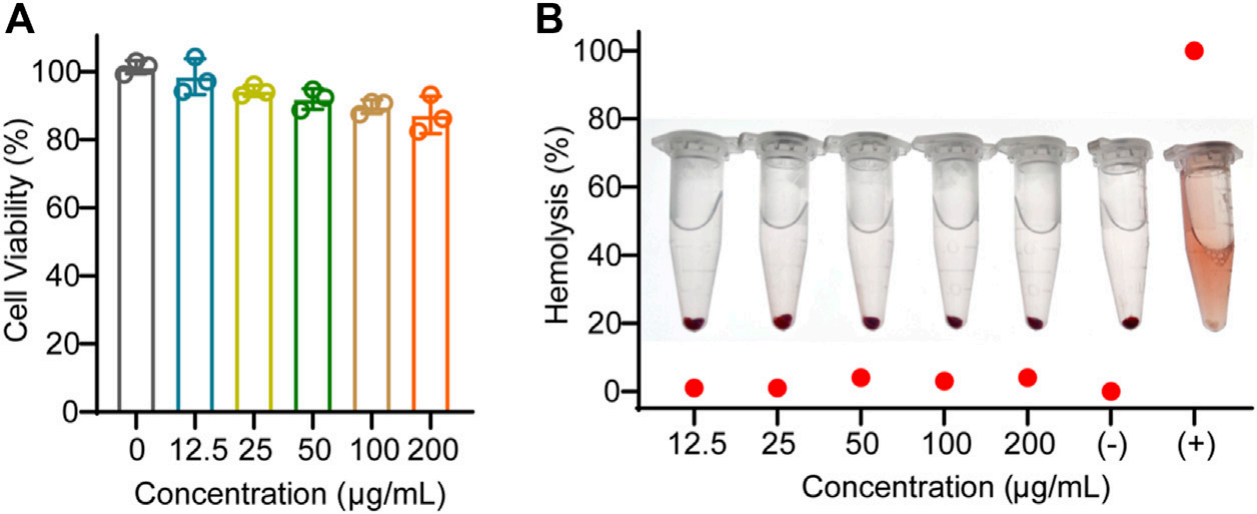
Biosafety evaluation of EV_Aloe._ **(A)** Macrophage viability against different concentrations of EV_Aloe_ treatment. **(B)** Blood hemolytic test of different concentrations of EV_Aloe_. Red blood cells were treated with a series of concentrations of TEV_Aloe_. Erythrocytes treated with PBS (0% hemolysis) were used as positive controls, and deionized water (100% hemolysis) was used as negative controls. N = 3, biologically independent replicates. Representative images per treatment group are shown.

To evaluate the biosafety of EV_Aloe_, we conducted cell viability and hemolysis assays. The EV_Aloe_ exhibited minimal toxicity to macrophages at dosages up to 200 μg/mL (Figure 2A). Hence, we set the EV_Aloe_ concentration below 200 μg/mL for assessing macrophage uptake and polarization modulation in our cellular study, considering that higher concentrations might induce cellular toxicity and complicate immunological responses. Hemolysis tests conducted on red blood cells incubated with various concentrations of EV_Aloe_ revealed no observable hemolysis within a wide range of EV_Aloe_ concentrations (Figure 2B).

### Uptake of EV_Aloe_ by macrophages through phagocytosis

**FIGURE 3:**
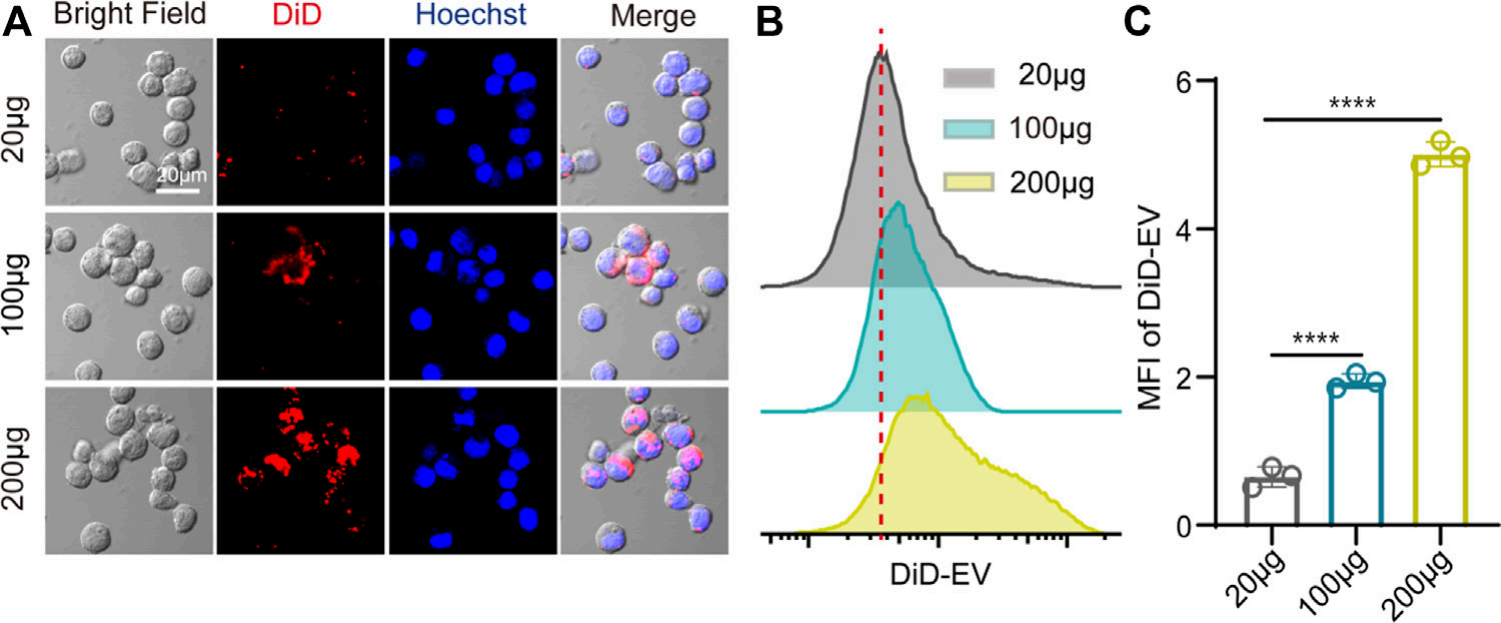
Cellular uptake analysis of EV_Aloe_ by the macrophage cell. **(A)** Cellular uptake of DiD-loaded EV with different doses of EV_Aloe_ after 4-h incubation with macrophages (Hoechst; blue), as assessed by confocal microscopy. White scale bars: 20 μm. **(B, C)** DiD-positive rates of macrophages cocultured with the DiD-labeled EV_Aloe_ for 4 h, analyzed by flow cytometry (n = 3). Representative images per treatment group are shown. The data are presented as the means ± SD. Statistical significance was calculated by one-way ANOVA with Tukey’s multiple comparisons test, *p < 0.05, **p < 0.01, ***p < 0.001, and ****p < 0.0001; ns denotes no significant difference.

To evaluate the impact of EV_Aloe_ on macrophage immune activation, we first assessed macrophage uptake and internalized EV_Aloe_. Employing DID-labeled EV_Aloe_, we visualized the phagocytosis and internalization of EV_Aloe_ on macrophages. Immunofluorescence imaging revealed a dose-dependent increase in EV_Aloe_ within macrophages (Figure 3A). Additionally, similar outcomes were obtained through flow cytometry analysis, where the fluorescence signal of EV_Aloe_ was notably higher in the group incubated with 200 μg EV_Aloe_ than in other groups, indicating a dose-dependent increase in EV_Aloe_ internalization by macrophages (Figures 3B, C). Importantly, as mentioned earlier, varying concentrations of EV_Aloe_ showed no evident cytotoxic effects on macrophages. Together, these results suggest that EV_Aloe_ can be engulfed and internalized by murine macrophages.

### Macrophage polarization induced by EV_Aloe_

**FIGURE 4:**
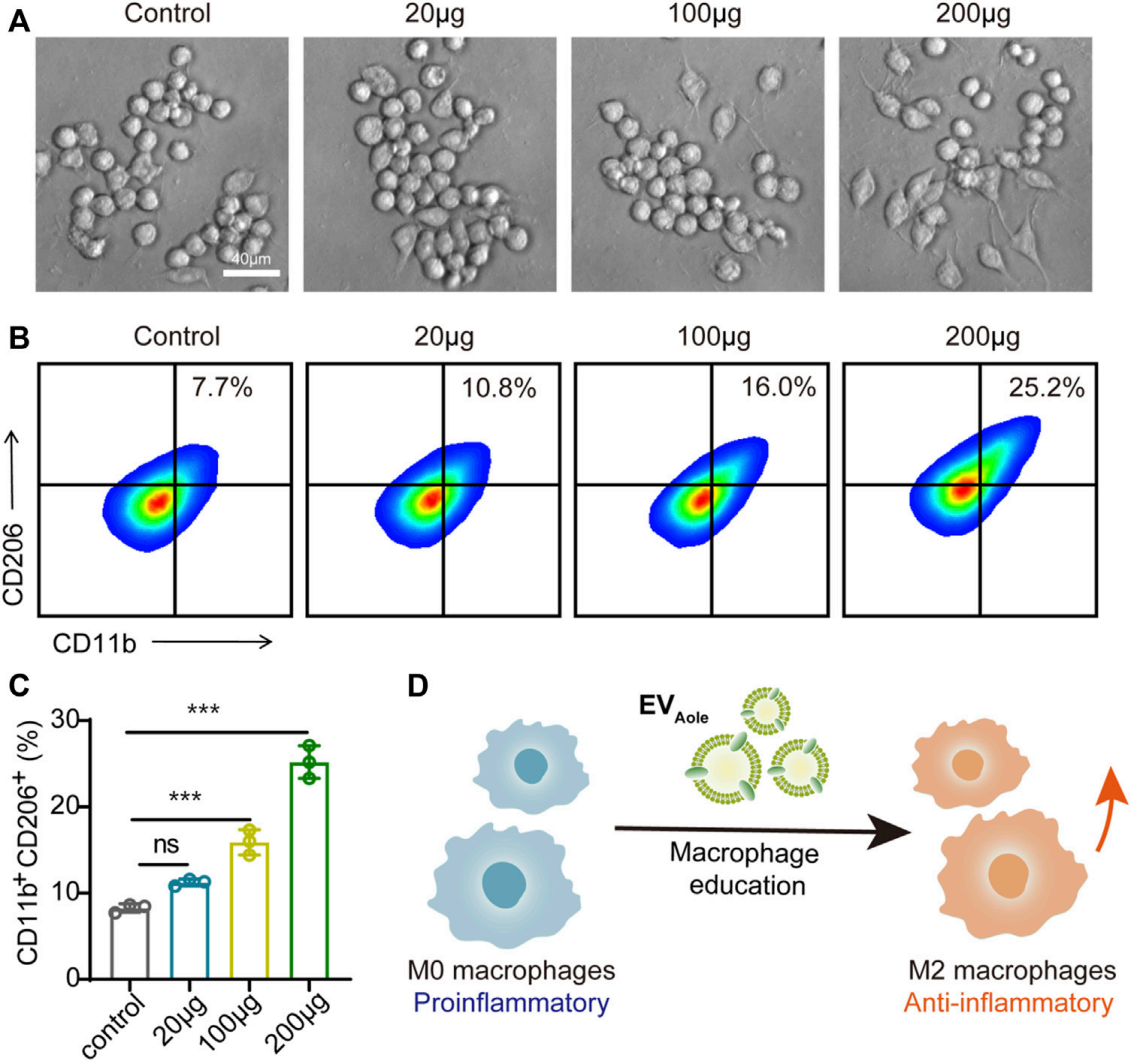
EV_Aloe_ facilitates macrophage phenotype reprogrammed. **(A)** Morphological changes in macrophages after different concentrations of EV_Aloe_ treatments. Representative images per treatment group are shown. **(B, C)** Flow cytometry images **(B)** and the corresponding quantification analysis **(C)** of CD11b^+^CD206^+^ M2 macrophages after incubation with EV_Aloe_ for 24 h. **(D)** Scheme of the EV_Aloe_ stimulation in the polarization of macrophages. N = 3, biologically independent samples. Representative images per treatment group are shown. The data are presented as the means ± SD. Statistical significance was calculated by one-way ANOVA with Tukey’s multiple comparisons test, *p < 0.05, **p < 0.01, ***p < 0.001, and ****p < 0.0001; ns denotes no significant difference.

Regarding the preceding experiments, we confirmed the internalization and uptake of EV_Aloe_ by murine macrophages. Subsequently, we assessed the impact of varying EV_Aloe_ concentrations on the polarization capacity and phenotypic alterations in macrophages. Bright-field microscopy showed a notable transformation in macrophage morphology following EV_Aloe_ treatment, particularly treated with higher EV_Aloe_ concentrations, inducing more pronounced alterations in cell shape (Figure 4A). Additionally, flow cytometry analysis of primary macrophages treated with diverse EV_Aloe_ dosages revealed the most substantial percentage of M2-like tumor-associated macrophages (TAMs) within the group exposed to 200 μg of EV_Aloe_ (Figures 4B, C). These observations suggest that EV_Aloe_ exhibits remarkable and dose-dependent immunomodulatory attributes, effectively steering macrophages toward an M2 immune-activating phenotype (Figure 4C).

### Clinical bronchoalveolar lavage fluid treatment

**FIGURE 5:**
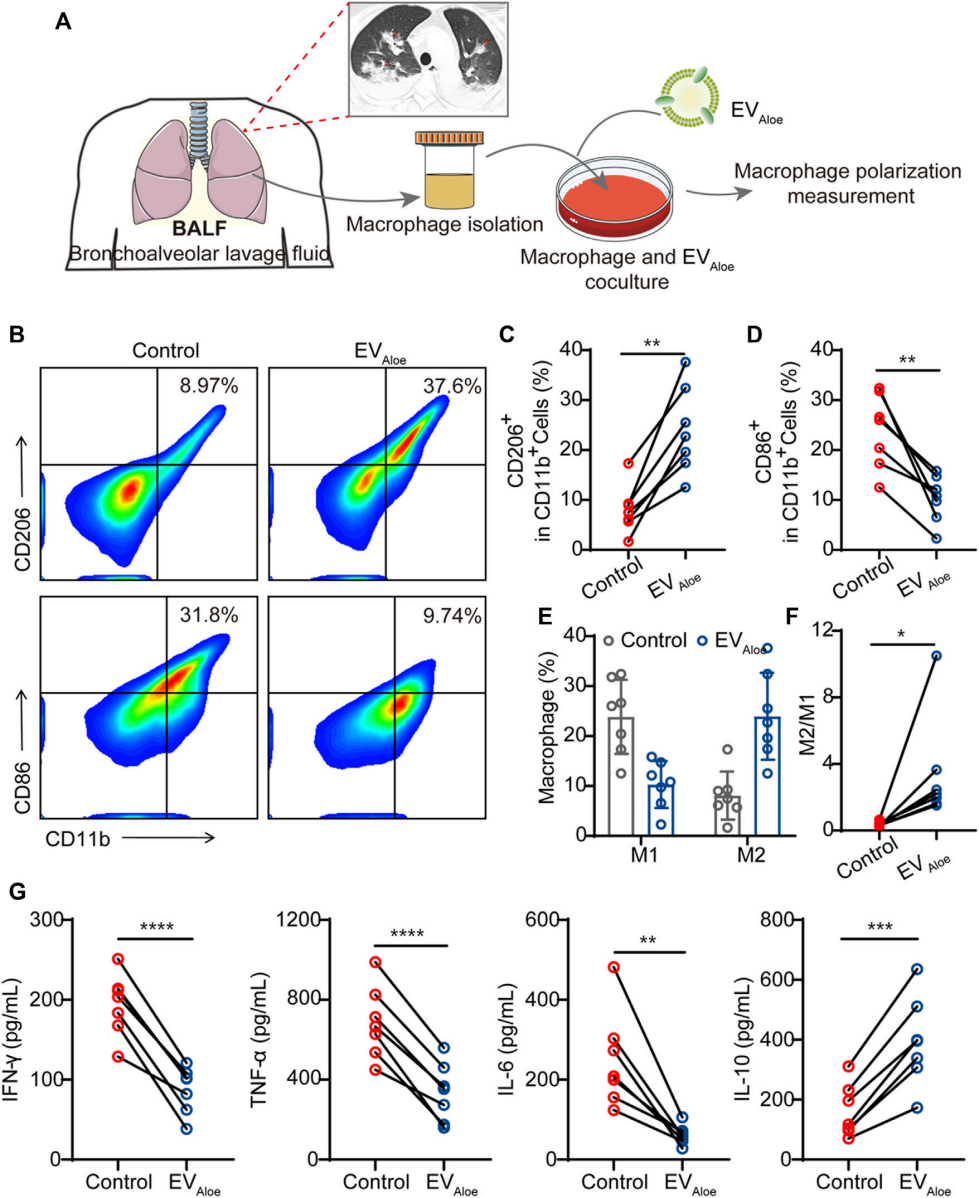
Immunological effects of EV_Aloe_ on the BALF. **(A)** Schematic design of the clinical study. EV_Aloe_ was selected to improve the immunosuppressive microenvironment of the BALF. **(B–D)** Representative flow cytometry images **(B)** and the quantification analysis of human macrophage polarization induced by EV_Aloe_. Human macrophage repolarization by EV_Aloe_ based on CD206 **(C)** and CD86 **(D)** expression. TAMs of the classical activation M2 phenotype highly expressed CD86 and downregulated the expression of M1-phenotype CD206 proteins (gated on CD11b+ cells) (n = 7 biological replicates). Representative images per treatment group are shown. **(E, F)** Percentage of M1-like and M2-like macrophages and relative quantification of M1/M2 **(F)** in BALF treated with EV_Aloe_ (n = 7). **(G)** Concentrations of cytokines in RAW 264.7 cell supernatants after incubation with the EV_Aloe_ groups for 24 h. The levels of IFN-γ, TNF-α, IL-6, and IL-10 were analyzed using the corresponding specific ELISA kits. N = 7 biologically independent replicates. The data are presented as the means ± SD. Statistical significance was calculated by one-way ANOVA with Tukey’s multiple comparison test, *p < 0.05, **p < 0.01, ***p < 0.001, and ****p < 0.0001; ns denotes no significant difference.

To assess the translational viability of murine macrophages influenced by EV_Aloe_, we conducted a clinical feasibility study employing clinical bronchoalveolar lavage fluid (BALF) as a representative model (Figure 5A). Compared with lung biopsy, BALF is safer and less invasive, with few complications, and the resulting sample is larger than the source bronchus and multiple lung lobes (Mondoni et al., 2022). The information gained from BALF-EVs is regarded to be a complement to lung biopsy pathology (Zareba et al., 2021). To delve into this, we gathered BALF samples from bacterial pneumonia patients (n = 7), supported by confirmed clinical images (Figure 5A). Our investigation focused on discerning the immune impact of EV_Aloe_ on macrophage cells within BALF under *ex vivo* conditions. Flow cytometry analysis of macrophages after EV_Aloe_ incubation showcased a substantial increase in the expression levels of M2-associated surface markers compared to the untreated BALF control (Figures 5B–D). Simultaneously, a correlated decrease in the expression of M1-related protein markers was observed (Figure 5E). These discernible alterations in polarization biomarkers were further authenticated by quantifying the M1/M2 ratio (Figure 5F), signaling the effective repolarization of macrophages due to EV_Aloe_ treatment. Furthermore, employing enzyme-linked immunosorbent assay (ELISA) to evaluate the inflammatory cytokine profile changes in BALF revealed increased levels of inflammatory cytokines in pristine pleural effusion across all samples, aligning with previous clinical observations of immune BALF (Figure 5G). However, upon EV_Aloe_ treatment, a significant increase in anti-inflammatory cytokines evidently indicated the efficacy of the treatment. In concert, these results validate the substantial potential of EV_Aloe_ for clinical research and its profound impact on immune modulation.

## Conclusion

In conclusion, we successfully isolated and purified EV_Aloe_ with the capacity to reprogram the immune phenotype of macrophages by consecutive centrifugation and ultracentrifugation. Characterization of the prepared EV_Aloe_ revealed its possession of typical features akin to conventional extracellular vesicles. Additionally, hemolysis and cytotoxicity assays validated the robust biosafety of our EV_Aloe_, demonstrating its ability to repolarize pro-inflammatory macrophages into an anti-inflammatory phenotype. Clinical assessments further confirmed that EV_Aloe_ effectively reduces inflammation levels and promotes tissue repair. Our findings demonstrate that EV_Aloe_, through cellular engulfment and internalization, can reprogram pro-inflammatory macrophages toward an anti-inflammatory phenotype, attenuating excessive inflammatory responses and facilitating tissue repair. We propose aloe-derived EVs as a highly efficient, safe, and immensely promising macrophage polarization agent for treating acute lung injury induced by bacterial pneumonia.

## Materials and methods

### Preparation and characterization of EV_Aloe_

EV_Aloe_ was isolated from aloe (bought from the Curacao aloe base of Kangyun Biological Company, Yunnan Province, China) juice by differential centrifugation and then purified using sucrose gradient centrifugation methods. In brief, the aloe was washed with deionized water and then homogenized using a blender. The mixtures were first consecutively centrifuged at 500 *g* for 10 min, 3,000 g for 10 min and 3,000 g for 30 min, and then, 10,000 g for 1 h to deplete large fibers and cell debris, and then, the supernatant was ultracentrifuged at 150,000 g for 2 h. We resuspended the obtained pellet of EV_Aloe_ in PBS and stored the solution at −80 °C until further use. For characterization of EV_Aloe_, the particle sizes of EV_Aloe_ were characterized by NTA (Particle Metrix ZetaView, Germany). After screening of size, EV_Aloe_ was prepared for TEM imaging; 10 μL EV_Aloe_ was deposited onto the surface of a formvar-coated copper grid, 1% uranyl acetate was then added for 15 s twice, and the sample was allowed to dry for subsequent imaging. The EV_Aloe_ protein expression was analyzed by SDS-PAGE, the concentrations of which were quantified based on protein concentration using Bicinchoninic Acid Protein Assay (KeyGEN BioTECH) following the manufacturer’s protocol. Loading samples were prepared with 20 µg of protein per well. After the proteins in the loading samples were denatured for 10 min at 95°C, the loading samples were analyzed by SDS-PAGE in a Stain-Free™ Precast Gel (Bio-Rad #4568094). Phase contrast images were captured using an inverted microscope (Olympus CX41, Japan), and fluorescent images were captured by laser confocal microscopy (FV1000MPE, Olympus).

### Biosafety test

To measure the cytotoxicity of EV_Aloe_ *in vitro*, RAW 264.7 cells were incubated with different concentrations of EV_Aloe_ for 24 h. The cell viability was evaluated by using a CCK-8 assay kit (BS350B, Biosharp). Furthermore, to assess the blood compatibility of EV_Aloe_, it was evaluated by hemolysis assay. In brief, pure 0.3 mL red blood cells were dispersed in 6 mL normal saline. Then, 0.1 mL of blood red blood cells were co-incubated with different concentrations of EV_Aloe_ (12.5 μg/mL, 25 μg/mL, 50 μg/mL, 100 μg/mL, and 200 μg/mL) at 37°C for 3 h. Distilled water and saline were regarded as the control. The mixtures were centrifuged, and then, the supernatant was measured at an absorbance of 540 nm. The hemolysis rate was calculated as follows:

$$\text{Hemolysis }\left(\%\right)=\left[A\left({\text{EV}}_{\text{Aloe}}\right)-A\left(\text{Negative}\right)/A\left(\text{Positive}\right)-A\left(\text{Negative}\right)\right]\times 100\%.$$

### *In vitro* macrophage uptake of EV_Aloe_

EV_Aloe_ was stained with 0.5 µM DiD far-red fluorescent probe (C1039, Beyotime) according to the manufacturer’s protocol. The RAW 264.7 macrophage cells were seeded into confocal dishes, and the different concentrations of EV_Aloe_ (20 μg, 100 μg, and 200 µg) were added for 4 h at 37 °C. Then, the cells were stained with the nucleus with the Hoechst 33258 (C1011, Beyotime). Laser confocal microscopy was used to present the stained cells (FV1000MPE, Olympus). Furthermore, the cells were then centrifuged at 500 *g* for 3 min and resuspended in PBS for further flow cytometry analysis. Fluorescent signals were assessed using a NovoCyte FACS flow cytometer (ACEA Biosciences, Inc.), and data were analyzed using FlowJo software.

### Macrophage polarization

To perform *in vitro* macrophage repolarization experiments, the initial M0 macrophages were treated with different concentrations of EV_Aloe_ (20 μg, 100 μg, and 200 µg) for 12 h at 37°C. Afterward, the macrophages were harvested and stained with anti-mouse CD11b-APC/Cyanine7 (BioLegend, Cat. No. 101226, clone M1/70) and anti-mouse CD206-PE (BioLegend, Cat. No. 141706, clone C068C2) and then subjected to flow cytometry. Fluorescent signals were detected using a NovoCyte FACS flow cytometer (ACEA Biosciences, Inc.), and data were analyzed using FlowJo software.

### Flow cytometry analysis of clinical BALF treatment

To examine macrophage phenotypic changes in the BALF, macrophages from BALF were separated via magnetic-activated cell sorting (MACS) using magnetic beads. For flow cytometry analysis, the macrophages after EV_Aloe_ treatment were fixed, permeabilized, and stained with anti-human CD11b-Percp/Cyanine5.5 (BioLegend, Cat. No. 301327, clone ICRF44) and M1 macrophage marker (anti-human CD80-PE, BioLegend, Cat. No. 305207, clone 2D10) and M2 macrophage marker (anti-human CD206-APC monoclonal Abs, BioLegend, Cat. No. 321109, clone 15-2) for flow analysis. All data were analyzed using FlowJo.

### Cytokine analysis

An ELISA kit was used to measure the concentrations of inflammatory cytokines and chemokines according to the manufacturer’s instructions. The macrophages from BALF were co-incubated with EV_Aloe_ for 24 h; then, the supernatant was collected for the detection of macrophage-related cytokines, such as the pro-inflammatory phenotype (IL-10, TNF-α, IFN-γ, and IL-6).

## Data Availability

The original contributions presented in the study are included in the article/Supplementary Material, further inquiries can be directed to the corresponding authors.

## References

[B1] Alvarez-Lerma F. (1996). Modification of empiric antibiotic treatment in patients with pneumonia acquired in the intensive care unit. Intensive Care Med. 22 (5), 387–394. 10.1007/BF01712153 8796388

[B2] Boccia E. Alfieri M. Belvedere R. Santoro V. Colella M. Del G. P. (2022). Plant hairy roots for the production of extracellular vesicles with antitumor bioactivity. Commun. Biol. 5 (1), 848. 10.1038/s42003-022-03781-3 35987960 PMC9392725

[B3] Braverman J. Monk I. R. Ge C. Westall G. P. Stinear T. P. Wakim L. M. (2022). *Staphylococcus aureus* specific lung resident memory cd4(+) th1 cells attenuate the severity of influenza virus induced secondary bacterial pneumonia. Mucosal Immunol. 15 (4), 783–796. 10.1038/s41385-022-00529-4 35637249 PMC9148937

[B4] Cilloniz C. Ewig S. Polverino E. Marcos M. A. Esquinas C. Gabarrus A. (2011). Microbial aetiology of community-acquired pneumonia and its relation to severity. Thorax 66 (4), 340–346. 10.1136/thx.2010.143982 21257985

[B5] Colombo M. Raposo G. Thery C. (2014). Biogenesis, secretion, and intercellular interactions of exosomes and other extracellular vesicles. Annu. Rev. Cell Dev.Biol. 30, 255–289. 10.1146/annurev-cellbio-101512-122326 25288114

[B6] Dad H. A. Gu T. W. Zhu A. Q. Huang L. Q. Peng L. H. (2021). Plant exosome-like nanovesicles: emerging therapeutics and drug delivery nanoplatforms. Mol. Ther. 29 (1), 13–31. 10.1016/j.ymthe.2020.11.030 33278566 PMC7791080

[B7] Gonzalez-Juarbe N. Gilley R. P. Hinojosa C. A. Bradley K. M. Kamei A. Gao G. (2015). Pore-forming toxins induce macrophage necroptosis during acute bacterial pneumonia. PLoS Pathog. 11 (12), e1005337. 10.1371/journal.ppat.1005337 26659062 PMC4676650

[B8] Hussell T. Bell T. J. (2014). Alveolar macrophages: plasticity in a tissue-specific context. Nat. Rev. Immunol. 14 (2), 81–93. 10.1038/nri3600 24445666

[B9] Ju S. Mu J. Dokland T. Zhuang X. Wang Q. Jiang H. (2013). Grape exosome-like nanoparticles induce intestinal stem cells and protect mice from dss-induced colitis. Mol. Ther. 21 (7), 1345–1357. 10.1038/mt.2013.64 23752315 PMC3702113

[B10] Kim M. K. Choi Y. C. Cho S. H. Choi J. S. Cho Y. W. (2021). The antioxidant effect of small extracellular vesicles derived from aloe vera peels for wound healing. Tissue Eng. Regen. Med. 18 (4), 561–571. 10.1007/s13770-021-00367-8 34313971 PMC8325744

[B11] Lambrecht B. N. (2006). Alveolar macrophage in the driver's seat. Immunity 24 (4), 366–368. 10.1016/j.immuni.2006.03.008 16618595

[B12] Magiorakos A. P. Srinivasan A. Carey R. B. Carmeli Y. Falagas M. E. Giske C. G. (2012). Multidrug-resistant, extensively drug-resistant and pandrug-resistant bacteria: an international expert proposal for interim standard definitions for acquired resistance. Clin. Microbiol. Infect. 18 (3), 268–281. 10.1111/j.1469-0691.2011.03570.x 21793988

[B13] Mondoni M. Rinaldo R. F. Carlucci P. Terraneo S. Saderi L. Centanni S. (2022). Bronchoscopic sampling techniques in the era of technological bronchoscopy. Pulmonology 28 (6), 461–471. 10.1016/j.pulmoe.2020.06.007 32624385

[B14] Monteith A. J. Miller J. M. Maxwell C. N. Chazin W. J. Skaar E. P. (2021). Neutrophil extracellular traps enhance macrophage killing of bacterial pathogens. Sci. Adv. 7 (37), eabj2101. 10.1126/sciadv.abj2101 34516771 PMC8442908

[B15] Ott S. R. Hauptmeier B. M. Ernen C. Lepper P. M. Nuesch E. Pletz M. W. (2012). Treatment failure in pneumonia: impact of antibiotic treatment and cost analysis. Eur. Resp. J. 39 (3), 611–618. 10.1183/09031936.00098411 21965229

[B16] Peng L. H. Wang M. Z. Chu Y. Zhang L. Niu J. Shao H. T. (2020). Engineering bacterial outer membrane vesicles as transdermal nanoplatforms for photo-trail-programmed therapy against melanoma. Sci. Adv. 6 (27), eaba2735. 10.1126/sciadv.aba2735 32923586 PMC7455490

[B17] Shi T. Denouel A. Tietjen A. K. Lee J. W. Falsey A. R. Demont C. (2020). Global and regional burden of hospital admissions for pneumonia in older adults: a systematic review and meta-analysis. J. Infect. Dis. 222 (Suppl. 7), S570–S576. 10.1093/infdis/jiz053 30849172

[B18] Torres A. Niederman M. S. Chastre J. Ewig S. Fernandez-Vandellos P. Hanberger H. (2017). International ers/esicm/escmid/alat guidelines for the management of hospital-acquired pneumonia and ventilator-associated pneumonia: guidelines for the management of hospital-acquired pneumonia (hap)/ventilator-associated pneumonia (vap) of the european respiratory society (ers), european society of intensive care medicine (esicm), european society of clinical microbiology and infectious diseases (escmid) and asociacion latinoamericana del torax (alat). Eur. Resp. J. 50 (3), 1700582. 10.1183/13993003.00582-2017 28890434

[B19] van der Poll T. van de Veerdonk F. L. Scicluna B. P. Netea M. G. (2017). The immunopathology of sepsis and potential therapeutic targets. Nat. Rev. Immunol. 17 (7), 407–420. 10.1038/nri.2017.36 28436424

[B20] Wang B. Zhuang X. Deng Z. B. Jiang H. Mu J. Wang Q. (2014). Targeted drug delivery to intestinal macrophages by bioactive nanovesicles released from grapefruit. Mol. Ther. 22 (3), 522–534. 10.1038/mt.2013.190 23939022 PMC3944329

[B21] Wen W. Su W. Tang H. Le W. Zhang X. Zheng Y. (2020). Immune cell profiling of covid-19 patients in the recovery stage by single-cell sequencing. Cell Discov. 6, 31. 10.1038/s41421-020-0168-9 32377375 PMC7197635

[B22] Xu X. H. Yuan T. J. Dad H. A. Shi M. Y. Huang Y. Y. Jiang Z. H. (2021). Plant exosomes as novel nanoplatforms for microrna transfer stimulate neural differentiation of stem cells *in vitro* and *in vivo* . Nano Lett. 21 (19), 8151–8159. 10.1021/acs.nanolett.1c02530 34586821

[B23] Zareba L. Szymanski J. Homoncik Z. Czystowska-Kuzmicz M. (2021). Evs from balf-mediators of inflammation and potential biomarkers in lung diseases. Int. J. Mol. Sci. 22 (7), 3651. 10.3390/ijms22073651 33915715 PMC8036254

[B24] Zhang D. Guo R. Lei L. Liu H. Wang Y. Wang Y. (2021). Frontline science: covid-19 infection induces readily detectable morphologic and inflammation-related phenotypic changes in peripheral blood monocytes. J. Leukoc. Biol. 109 (1), 13–22. 10.1002/JLB.4HI0720-470R 33040384 PMC7675546

[B25] Zhang M. Viennois E. Prasad M. Zhang Y. Wang L. Zhang Z. (2016). Edible ginger-derived nanoparticles: a novel therapeutic approach for the prevention and treatment of inflammatory bowel disease and colitis-associated cancer. Biomaterials 101, 321–340. 10.1016/j.biomaterials.2016.06.018 27318094 PMC4921206

